# Possible Contribution of Multiple Sclerosis Pathology to Treatment‐Resistant Schizophrenia: A Case Study

**DOI:** 10.1002/npr2.70158

**Published:** 2026-08-06

**Authors:** Sho Miura, Atsuko Nagaoka, Mizuki Hino, Risa Shishido, Masataka Hatano, Yuto Hosogai, Kazusa Miyahara, Yuta Oba, Kimihiko Kaneko, Yasuto Kunii, Hiroaki Tomita

**Affiliations:** ^1^ Department of Psychiatry Tohoku University Hospital Sendai Miyagi Japan; ^2^ Department of Neuropsychiatry, School of Medicine Fukushima Medical University Fukushima Japan; ^3^ Department of Disaster Psychiatry, International Research Institute of Disaster Science Tohoku University Sendai Japan; ^4^ Department of Neurology Tohoku University Hospital Sendai Miyagi Japan; ^5^ Department of Psychiatry, Graduate School of Medicine Tohoku University Sendai Miyagi Japan

**Keywords:** multiple sclerosis, psychosis, treatment‐resistant schizophrenia

## Abstract

Multiple sclerosis (MS) can present with neurological and psychiatric symptoms that may overlap with those of schizophrenia, making differentiation difficult particularly because their typical onset ages are similar. We present the case of a woman in her 50s who developed persecutory delusions and a subjective sense of being under surveillance in Year X‐31 and was diagnosed with schizophrenia. Despite the long‐term antipsychotic treatment, the patient's symptoms persisted and worsened. In Year X‐1, brain magnetic resonance imaging revealed multiple white matter lesions, and then cerebrospinal fluid analysis revealed oligoclonal bands, leading to a diagnosis of MS. Treatment with ofatumumab was initiated. Owing to persistent psychiatric symptoms, clozapine was administered, which showed partial effectiveness. This case highlights the clinical overlap between schizophrenia and MS and suggests that comorbid MS pathology may be clinically relevant in the context of treatment resistance and cognitive impairment. Similarities in symptoms and disease course raise the possibility that a subset of patients with schizophrenia may share pathophysiological features with MS. In addition, postmortem studies, including our previous and current observations, provide hypothesis‐generating support for partially overlapping MS‐like changes in some patients with schizophrenia. Clinicians should consider the possibility of comorbid MS in patients with treatment‐resistant schizophrenia, particularly when atypical clinical or neurological features are present.

## Abstract 2

A case of multiple sclerosis detected by brain MRI during evaluation for modified electroconvulsive therapy in the long‐term course of schizophrenia. This case discusses the clinical overlap between multiple sclerosis pathology and treatment‐resistant schizophrenia.

## Graphical abstract

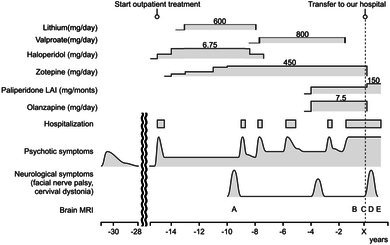

## 1 Introduction

Multiple sclerosis (MS) is characterized by acute or subacute onset of neurological symptoms, which frequently include muscle weakness, gait disturbance, sensory impairment, and visual impairment. MS presents with a variety of clinical symptoms, including extrapyramidal symptoms, intention tremors, bladder–rectum dysfunction, and seizures, which occur multiple times and undergo repeated exacerbations and remissions [1]. MS is also associated with a variety of psychiatric symptoms, including depressed mood, fatigue, suicidal ideation, and cognitive decline [2]. Two to three percent of patients exhibit psychotic symptoms characterized by persecutory delusions and auditory hallucinations, such as hearing one's name being called, and may exhibit symptoms similar to schizophrenia [3]. MS most commonly occurs at approximately 30 years [1], which is close to that at which schizophrenia occurs, in the late teens and 30s. Therefore, it is important to consider MS in the differential diagnosis of schizophrenia. When psychiatric symptoms are prominent in the early stages of MS and neurological symptoms are not prominent, or when imaging examinations do not reveal typical abnormal findings in the cerebral white matter, it can be difficult to distinguish between MS and schizophrenia. Additionally, MS occurring during the course of schizophrenia may modify psychiatric symptoms and affect the development of treatment resistance. Here, we discuss a case in which MS was accidentally discovered during the long‐term of treatment‐resistant schizophrenia (TRS).

## 2 Case Presentation

**FIGURE 1:**
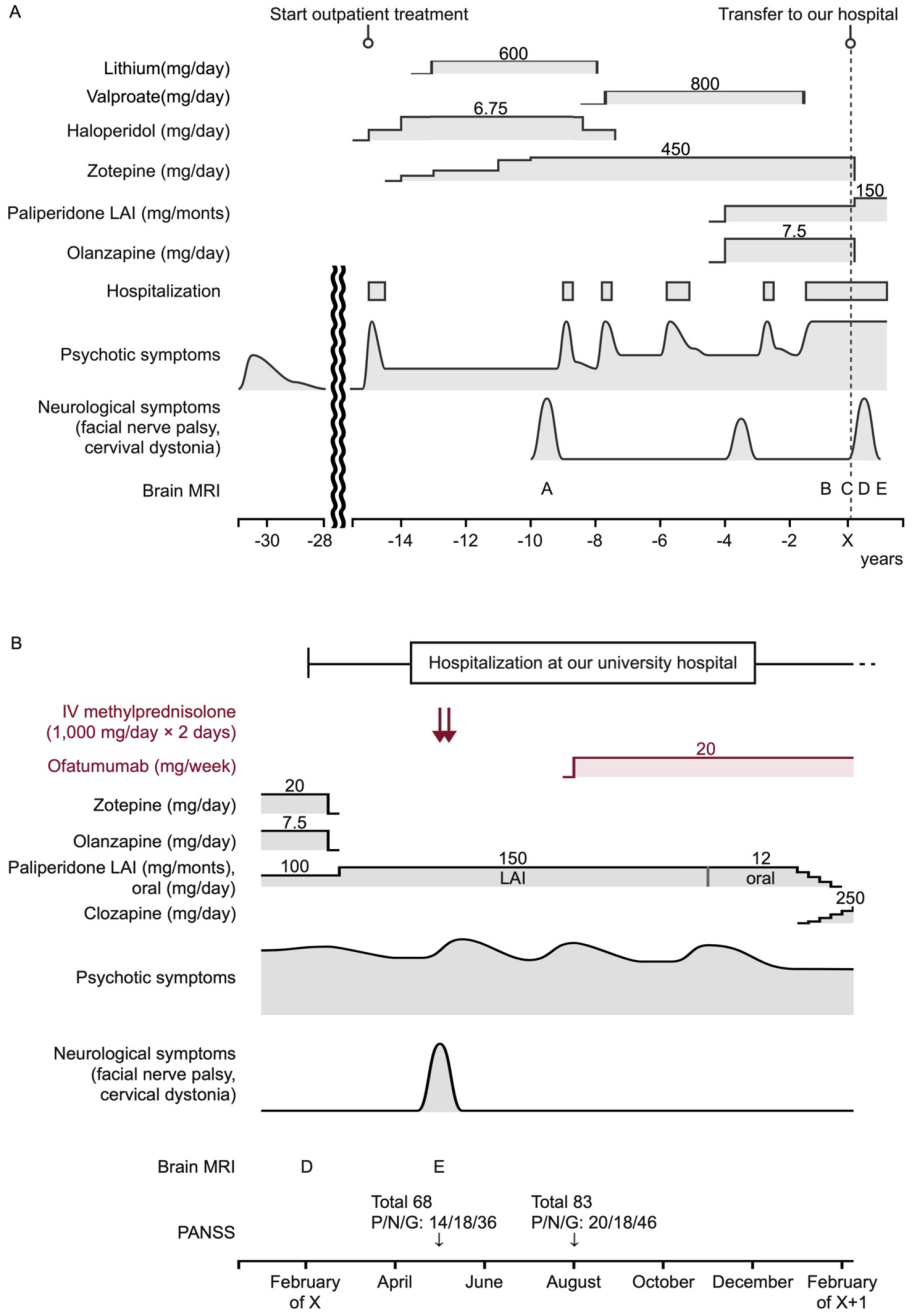
Progress chart. (A) The patient's clinical course from year X‐31, when she began to complain of feelings of being watched and delusions of persecution, until her transfer to our hospital in year X. She first visited a psychiatrist on X‐28, and her symptoms improved thereafter. After approximately 13 years of no treatment, she was admitted to a psychiatric hospital in X‐15 due to hallucinations and delusions and continued to be seen as an outpatient after discharge. After the onset of left facial nerve paralysis on X‐10, her psychiatric symptoms became unstable, and she was repeatedly hospitalized. Brain magnetic resonance imaging (MRI) performed at X‐1 suggested multiple sclerosis, and this prompted transfer to our hospital in year X for further evaluation. (B) The clinical course of inpatients at our institution from the date of transfer. Long‐acting injectable (LAI) paliperidone was transitioned to oral paliperidone to allow gradual tapering during cross‐titration to clozapine. Positive and Negative Syndrome Scale (PANSS) scores are presented as “Total; P/N/G,” where P, N, and G denote the Positive, Negative, and General Psychopathology subscales, respectively. The time points labeled (A–E) correspond to the MRI examinations shown in Figure 2A–E. The curves labeled “Psychotic symptoms” and “Neurological symptoms” represent retrospective, clinician‐rated schematic estimations of relative symptom severity over time and are not based on standardized rating scales. Higher peaks indicate greater symptom severity, whereas lower levels indicate relatively milder symptoms. The labels for intravenous methylprednisolone and ofatumumab are shown in light red to distinguish the treatments administered for multiple sclerosis from other medications used primarily for schizophrenia‐related symptoms.

**FIGURE 2:**
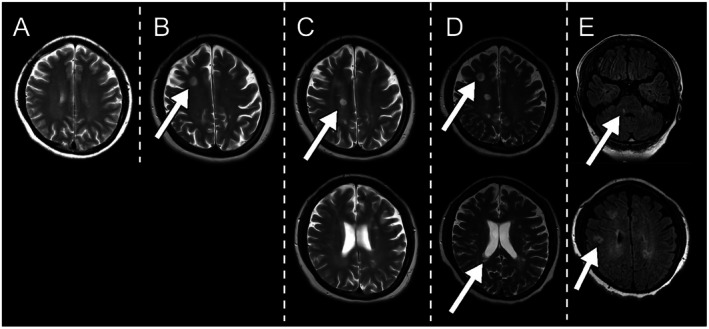
Brain magnetic resonance imaging (MRI). T2‐weighted image (A) in year X‐10 when the patient had left facial nerve paralysis and no lesions were found. T2‐weighted image (B) obtained during hospitalization at another hospital. High signal intensity was confirmed for the first time in the white matter surrounding the right lateral ventricle and the patient was placed under observation. Approximately 1 year later, T2‐weighted imaging (C) revealed a new lesion in the anterior part of the body of the right lateral ventricle. The patient was referred to our hospital's Department of Neurology, where a gadolinium‐enhanced T2‐weighted image (D) shows a lesion with an enhancing effect in the posterior part of the body of the right lateral ventricle. After the diagnosis of multiple sclerosis was confirmed, right peripheral facial nerve palsy was observed. Gadolinium‐enhanced fluid‐attenuated inversion recovery (FLAIR) MRI (E) revealed a new active lesion on the dorsal side of the pons. Arrow indicates a new lesion at the time of imaging.

**FIGURE 3:**
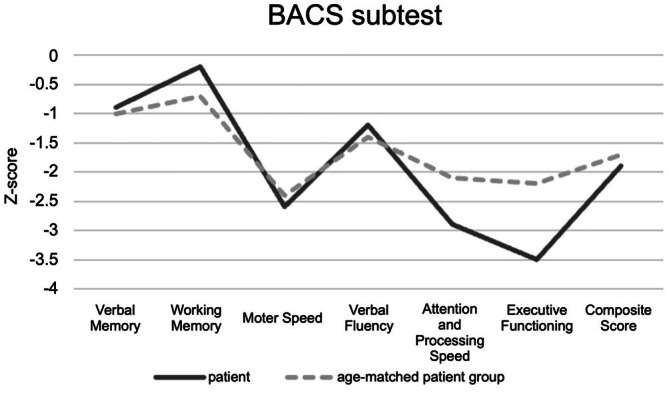
The Brief Assessment of Cognition in Schizophrenia (BACS) score of this case and the average BACS score of patients with schizophrenia of the same age. The solid line represents the BACS score of this patient. The dotted line represents the average BACS score of patients with schizophrenia of the same age. In this case, the scores for “Attention and Processing Speed” and “Executive Function” were significantly lower than the average for a schizophrenia group of the same age [4].

The patient was a woman in her 50s, born in Japan as the first of two siblings, with no family history of schizophrenia or MS. She had high academic achievement and entered a university in an urban area. Thirty‐one years before presentation (X‐31), shortly after enrollment, she developed persecutory delusions and a persistent sense of being watched, and withdrew from university. In X‐28 she attended a local psychiatric clinic and was diagnosed with atypical psychosis but discontinued follow‐up without medication. Although hallucinations and delusions subsequently diminished and her symptoms were relatively stable for periods, she became socially withdrawn. She engaged intermittently in short‐term employment; however, episodic exacerbations of psychotic symptoms led to frequent job discontinuation, preventing sustained employment, and eventually becoming socially isolated at home (Figure 1A). In X‐15 she re‐presented with hallucinations and delusions, was diagnosed with schizophrenia, and was hospitalized. Haloperidol was started, and lithium was added to manage mood instability and agitation, with transient improvement. The initial benefit of haloperidol was not sustained, and subsequent deterioration became more pronounced as medication non‐adherence emerged. Even during outpatient treatment, the patient did not sustain independent functioning. Family members accompanied her during all outpatient visits and provided continuous support in daily life. Home‐based occupational therapy was introduced to promote social functioning; however, no meaningful functional recovery was achieved. In X‐10 she developed left peripheral facial nerve palsy; brain MRI at that time showed no intracranial lesions (Figure 2A), and symptoms remitted after steroid therapy. According to both the patient and her family, no acute or transient neurological symptoms or prior neurological care episodes were noted prior to this episode; therefore, it was considered the first recognized neurological manifestation. Thereafter, psychiatric instability increased. Adherence was assessed based on documented medication refusal in clinical records, supervised administration during hospitalization, injection records for long‐acting injectable (LAI) paliperidone, and collateral information from family members. She refused medications (stating “I only want to take the medicine that I developed”), manifested delusions of poisoning, frequently contacted police, and exhibited food refusal. Haloperidol (up to 6.25 mg/day) was switched to zotepine (up to 450 mg/day) with only partial benefit. During hospitalization at another hospital, adherence to zotepine was ensured by supervised administration. Lithium was later replaced with valproic acid, which was also prescribed for mood instability and agitation but without clinical benefit. In X‐4 involuntary tongue and neck movements led to a neurology consultation and diagnosis of drug‐induced cervical dystonia. Medication non‐adherence worsened; paliperidone long‐acting injection (up to 150 mg intramuscularly every 4 weeks) was added alongside zotepine. During inpatient care, paliperidone LAI was administered according to schedule without missed doses. Olanzapine (up to 7.5 mg/day) was subsequently administered during supervised inpatient treatment; however, no significant improvement in the psychotic symptoms was observed. Taken together, the overall clinical course met TRS criteria. In March of X‐1, MRI (magnetic resonance imaging) obtained during hospitalization revealed a periventricular lesion adjacent to the right lateral ventricle (Figure 2B). In January X, follow‐up MRI performed as part of the pre‐evaluation for modified electroconvulsive therapy (mECT) revealed a new lesion in the anterior body of the right lateral ventricle (Figure 2C). Due to this unexpected finding, the patient was transferred to the Department of Psychiatry at our hospital in February X for further evaluation, including consultation with the Department of Neurology; therefore, mECT was not performed. After transfer to Department of Psychiatry at our hospital, the patient developed fecal ileus; all oral antipsychotics were stopped, and subsequent rigidity with slight creatine kinase elevation raised concern for neuroleptic malignant syndrome, so only paliperidone LAI was continued (Figure 1B). Neurology assessment with cerebrospinal fluid (CSF) analysis demonstrated oligoclonal bands; combined with MRI findings (Figure 2D), a diagnosis of MS was made. In May of X, she developed right peripheral facial nerve palsy with MRI evidence of an active MS lesion (Figure 2E). After the second episode of peripheral facial nerve palsy, forgetfulness became more prominent, suggesting an additional cognitive worsening in temporal association with MS disease activity. Steroid pulse therapy was initiated, but discontinued on day 2 due to marked psychomotor agitation, hallucinations, and self‐harm attempt, without observable improvement in psychotic symptoms. Steroid therapy was not administered thereafter. Ofatumumab (anti‐CD20) was begun for relapse prevention. Following the initiation of ofatumumab treatment, no further MS relapses or new neurological deficits were observed during follow‐up. Despite immunotherapy, persecutory delusions and emotional lability persisted. During hospitalization, she also showed periods of disorganized thinking with loosening of associations, leading to tangential and incoherent speech. The Positive and Negative Syndrome Scale (PANSS) was administered twice during hospitalization. In May of X‐1, the PANSS total was 68 (Positive 14, Negative 18, General Psychopathology 36), and in August of X‐1, the PANSS total was 84 (Positive 20, Negative 18, General Psychopathology 46), indicating persistently elevated positive and negative symptoms with worsening overall psychopathology despite ongoing treatment. Throughout the treatment at our institution, the patient's sensorium was consistently clear, and no episodes of delirium or fluctuating consciousness were observed. Clozapine was initiated due to poor response and intolerance to prior regimens, yielding partial improvement of affective instability but persistent delusions. Brief Assessment of Cognition in Schizophrenia (BACS) testing (Figure 3) showed marked deficits in attention and executive function relative to age‐matched schizophrenia cohorts [4], despite a history of high premorbid intellectual function.

## 3 Discussion

The present patient had been treated with antipsychotic drugs for schizophrenia since a young age for a long period. During hospitalization at another hospital, she received zotepine at doses of up to 450 mg/day and paliperidone LAI (150 mg every 4 weeks) to ensure adequate dosage, duration, and adherence. The patient was treated with other antipsychotics, including haloperidol. Despite adequately administered treatment, persistent persecutory delusions and hallucinations persisted, resulting in repeated hospitalizations. Therefore, the patient in the present case met these diagnostic criteria of TRS. In some patients, responsiveness to antipsychotic treatment diminishes over time, and relapses may occur despite ongoing maintenance treatment. Such breakthrough exacerbations can increase the relapse frequency and prolong periods of active psychotic symptoms, with cumulative adverse effects on functioning over time. Although this patient had a long history of treatment for schizophrenia, no head MRI or other examinations were performed at the time of onset of psychotic symptoms, and no intracranial lesions were found on MRI for the left facial nerve paralysis on X‐10. Simultaneously, the relationship observed in this case should be interpreted as associative rather than causal.

Following the initial diagnosis of psychosis, treatment was interrupted for 13 years. However, treatment was initiated on X‐15, and no exacerbation of symptoms occurred for 5 years. After the appearance of left facial nerve paralysis on X‐10, the patient was repeatedly admitted to and discharged from the hospital because of worsening psychiatric symptoms. The present case illustrates a clinical course characterized by long illness duration, repeated relapses with intermittent breakthrough psychotic exacerbations during ongoing antipsychotic treatment, cumulative antipsychotic exposure, progressive loss of social functioning, and marked cognitive impairment relative to high premorbid functioning, consistent with a gradual and sustained cognitive decline and a late progression toward TRS. MS was identified while further treatment options were being considered. When MS lesions became apparent on head MRI, the frequency of psychiatric symptom exacerbation and the length of hospitalization gradually increased. Cognitive decline, characterized mainly by attentional and executive dysfunction, has been reported to occur over time in MS [5]. In the present case, the BACS scores for these two domains were significantly lower than the average scores of age‐matched patients with schizophrenia [4]. While impairment of cognitive functions, including attention/processing speed and executive function, is a hallmark of schizophrenia, its unusual severity and temporal association with MS disease activity in this case suggest that MS‐related demyelination co‐occurred with the pre‐existing schizophrenia pathology, potentially exacerbating the cognitive burden and functional decline. However, because such findings are not specific to MS, they were interpreted together in broader clinical and neurological contexts rather than in isolation. Although the overall cognitive trajectory appeared gradual over the long course of schizophrenia, forgetfulness became more noticeable after the second episode of peripheral facial nerve palsy associated with active MS, suggesting that additional cognitive worsening was temporally related to MS disease activity. Therefore, cognitive impairment alone is not sufficient to suggest MS and should be interpreted in a broader clinical and neurological context, particularly when atypical neurological features, MRI/CSF abnormalities, or temporal relationships with MS activity or treatment are present. These findings suggest that the comorbid MS pathology might have modified the clinical presentation and complicated the response to antipsychotic treatment, potentially contributing to the development of a treatment‐resistant phenotype. In addition, corticosteroid pulse therapy initiated for active MS is temporally associated with marked psychomotor agitation, hallucinations, and self‐harming behavior, suggesting that MS‐directed treatment itself may psychiatrically destabilize the course and complicate the management of TRS. Further investigations of the potential association between MS lesions and antipsychotic intolerance are warranted.

MS is an inflammatory demyelinating disease in which chronic inflammation is caused by an autoimmune reaction in the central nervous system that destroys myelin and causes demyelination. Neuromyelitis optica, once considered a subtype of MS because of its similar clinical symptoms, presents with symptoms related to the optic nerve and spinal cord, and anti‐aquaporin 4 (AQP4) antibodies are involved in its pathology [6]. The discovery of autoantibodies in autoimmune encephalitis, the concept of autoimmune psychosis, and the presence of autoantibodies against the nervous system in patients with schizophrenia have drawn attention to the involvement of autoimmunity and autoantibody‐related mechanisms [7]. For example, a previous study suggested that anti‐NRXN1 autoantibodies found in the serum and CSF of patients with schizophrenia may be associated with social impairment and cognitive decline [8]. Oligodendrocyte dysfunction and abnormal myelination have also been reported to be involved in schizophrenia [9]. Collectively, these findings support the possibility of partially overlapping biological pathways in schizophrenia and MS, including neuroinflammation, glial involvement, and white matter pathology, rather than a single unified disease process. A study using mRNA data from postmortem brains of patients with schizophrenia found MS‐related pathways in a group of genes with a common brain expression pattern associated with cognitive impairment during life [10]. Analysis of a genome‐wide association study dataset of more than 20 000 individuals revealed genetic risk loci associated with both MS and schizophrenia [11]. In a comprehensive protein expression analysis using postmortem brains of patients with schizophrenia that we previously reported, AQP4 protein expression was decreased in the frontal cortex of patients with schizophrenia compared to non‐psychiatric controls [12]. Additional postmortem analyses further suggested that a subset of schizophrenia cases may show molecular features that partially overlap with those observed in MS; details are provided in the Supporting Information. These postmortem data were derived from independent schizophrenia samples and presented to provide a hypothesis‐generating mechanistic context. Importantly, the psychotic symptoms associated with MS are not necessarily identical to those of schizophrenia in phenomenology, longitudinal course, or treatment response, and these two disorders remain distinct diagnostic entities. MS is defined by objective evidence of inflammatory demyelination within the central nervous system, whereas schizophrenia is diagnosed based on a persistent psychotic disorder with characteristic functional impairment and enduring psychopathology. In treatment‐resistant schizophrenia, these overlapping biological themes may provide a framework for considering comorbid MS as a clinically relevant factor in selected cases.

## 4 Conclusion

Schizophrenia and MS are important differential diagnoses because they occur at similar ages and have similar clinical symptoms. In patients with TRS, particularly when prominent attentional and executive dysfunction is present in conjunction with atypical clinical or neurological features, the possibility of comorbid MS should be carefully considered. In addition to possible MS‐related pathology, the present case illustrates a clinical course characterized by long illness duration, repeated relapses, and cumulative antipsychotic exposure, which have been implicated in the progression of TRS. Studies using postmortem brains suggest that some patients with schizophrenia may show partially overlapping MS‐like pathologies. These observations provide a hypothesis‐generating framework for considering biological heterogeneity in schizophrenia.

## Author Contributions

S.M., A.N., and Y.K. conceived the study, conducted the literature review, and drafted and revised the manuscript. S.M., A.N., Y.O., K.K., and H.T. participated in the clinical management of patients. A.N., M. Hino, R.S., M. Hatano, Y.H., Y.K., and H.T. collected postmortem brain samples and associated clinical information. A.N., M. Hino, R.S., M. Hatano, and Y.K. conducted RNA data analysis. K.M. generated the figures. All authors contributed to the final manuscript and approved its submission for publication.

## Funding

This work was supported in part by the Grant‐in‐Aid for Early‐Career Scientists from JSPS KAKENHI (grant number 24K18734); the Strategic Research Program for Brain Sciences from AMED (grant numbers JP25wm0625126); the Grants‐in‐Aid for Scientific Research (B) from JSPS KAKENHI (grant number 24K02376); the Grants‐in‐Aid for Scientific Research (C) from JSPS KAKENHI (grant number 25K19063); and the Collaborative Research Project of Brain Research Institute, Niigata University (grant number 25009).

## Ethics Statement

This study was approved by the Ethics Committees of Fukushima Medical University (approval number: 418 and 1685) and Tohoku University Hospital (approval number: 2025‐1‐139) and was conducted in accordance with the Declaration of Helsinki.

## Consent

Written informed consent was obtained from the patient for the publication of this case report.

## Conflicts of Interest

The authors declare no conflicts of interest.

## Supporting information


**Figure S1:** npr270158‐sup‐0001‐supinfo.zip. *AQP4* mRNA levels in the prefrontal cortex from 25 patients with schizophrenia. Three patients exhibited high *AQP4* expression levels (grouped into SZhigh). SZhigh, schizophrenia with high *AQP4* expression; SZlow, schizophrenia with low *AQP4* expression.
**Figure S2:** GFAP protein levels in the prefrontal cortex from 23 patients with schizophrenia. Patients in the SZhigh group tended to exhibit higher GFAP protein levels than those in the SZlow group. SZhigh group, schizophrenia with high *AQP4* expression; SZlow group, schizophrenia with low *AQP4* expression.
**Figure S3:** A pathway analysis of the top 6000 differentially expressed genes (DEGs) between SZhigh and SZlow groups. A bar plot shows the top 30 pathways that the DEGs were enriched in. The upregulated pathways in SZhigh group are shown in red, while the downregulated pathways in SZhigh group are shown in blue. DEGs were significantly enriched in pathway related to multiple sclerosis (highlighted in bold). SZhigh group, schizophrenia with high *AQP4* expression; SZlow, schizophrenia with low *AQP4* expression.
**Table S1:** Source data underlying Figures S1 and S2. This table provides anonymized AQP4 mRNA values, GFAP protein values in the prefrontal cortex, and SZhigh/SZlow group labels used for Figures S1 and S2. “NA” indicates that the GFAP protein value was not available.
**Table S2:** Processed DEG list underlying Figure S3. This table provides the processed DEG list used as input for Ingenuity Pathway Analysis (IPA). The column “logFC_SZhigh_vs_SZlow” corresponds to the original logFC value from the edgeR output; positive values indicate higher expression in the SZhigh group relative to the SZlow group.
**Table S3:** IPA canonical pathway output underlying Figure S3. This table provides the canonical pathway output from IPA, including pathway‐level statistics and associated molecules, where available.

## Data Availability

Anonymized processed source data underlying Figures S1–S3 are provided in Supporting Information. This file includes the individual AQP4 mRNA values and SZhigh/SZlow labels, the individual GFAP protein values and group labels, the processed DEG list used as input for Ingenuity Pathway Analysis, and the IPA canonical pathway output. The data presented in this study are available from the corresponding author upon reasonable request. However, we have not yet obtained explicit consent from the legal next of kin to make the full gene expression data publicly available. For this reason, we are unable to deposit the raw sequencing data in a public repository at this time.
