# Supplementary material for: Possible Contribution of Multiple Sclerosis Pathology to Treatment‐Resistant Schizophrenia: A Case Study
Source: Neuropsychopharmacol Rep. 2026 Aug 6;46(3):e70158. doi: 10.1002/npr2.70158 (PMC13445245; doi:10.1002/npr2.70158)
Supplement: Supplementary file 1 [file NPR2-46-e70158-s001.zip › npr270158-sup-0002-supinfo02.docx]

**Supplementary information**

**Additional postmortem analyses**

In an additional examination, we analyzed the mRNA expression levels of *AQP4* in the frontal cortex of 25 patients with schizophrenia using postmortem brains and found that *AQP4* mRNA expression was high in three of the 25 patients with schizophrenia (SZhigh group) (Supplementary Figure 1). In MS, reactive gliosis occurs, and AQP4 is expressed in astrocytes; therefore, it is assumed that AQP4 expression is increased in MS [1]. We compared the glial fibrillary acidic protein (GFAP) protein expression levels in the frontal cortex of the SZhigh group and 22 patients with schizophrenia and low *AQP4* mRNA expression (SZlow group) using a t-test. GFAP levels tended to be higher in the SZhigh group (P=0.07), suggesting an increase in astrocytes (Supplementary Figure 2). Pathway analysis was performed on the top 6,000 DEGs with different mRNA expression levels between the SZhigh and SZlow groups, and a significant accumulation of pathways related to MS was confirmed (Supplementary Figure 3).

**Detailed Method**

Postmortem brain samples from 25 patients with schizophrenia (SZ) were obtained from the Tohoku Postmortem Brain Bank and DNA Bank for Psychiatric Research (<https://www.fmu-bb.jp/english/index.html>)[2][3]. This study was approved by the ethics committees of Fukushima Medical University and the Tohoku University Graduate School of Medicine. All procedures were performed after obtaining written informed consent from the next of kin. Diagnoses of SZ were based on criteria from the fourth or fifth edition of the Diagnostic and Statistical Manual of Mental Disorders (DSM-IV or 5).

RNA isolation and mRNA sequencing were performed as previously described [2][3]. Briefly, total RNA was isolated from the prefrontal cortex (PFC) of frozen brains using an AllPrep DNA/RNA Mini Kit (Qiagen, Tokyo, Japan). RNA purity was evaluated by the RNA integrity number (RIN) using an Agilent 2200 TapeStation (Agilent, Santa Clara, CA). The poly(A)+ RNA fraction was isolated from total RNA, and fragmented mRNA was reverse-transcribed into double-stranded complementary DNA (ds-cDNA). The ds-cDNA fragments were processed to generate cDNA libraries, which were then subjected to paired-end 2 × 101 bp sequencing on a HiSeq 4000 platform (Illumina, Tokyo, Japan).

Differentially expressed genes (DEGs) between the high and low groups were detected using edgeR (version 4.0.16) [4], with sex as a covariate. We used the ratio of gene expression levels in patients in the SZ low group as a reference (SZ high group/SZ low group). Genes with p < 0.05 were considered DEGs. Because of the limited sample size, uncorrected p-values (<0.05) were used to generate a gene list for exploratory pathway analysis. DEG functions were examined by canonical pathway analysis using Ingenuity Pathway Analysis (IPA) [5].

Protein expression data for GFAP in the PFC were obtained from our previous study [6].

Supplementary Figure 1. *AQP4* mRNA levels in the prefrontal cortex from 25 patients with schizophrenia. Three patients exhibited high *AQP4* expression levels (grouped into SZhigh). SZhigh, schizophrenia with high *AQP4* expression; SZlow, schizophrenia with low *AQP4* expression.

Supplementary Figure 2. GFAP protein levels in the prefrontal cortex from 23 patients with schizophrenia. Patients in the SZhigh group tended to exhibit higher GFAP protein levels than those in the SZlow group. SZhigh group, schizophrenia with high *AQP4* expression; SZlow group, schizophrenia with low *AQP4* expression.

Supplementary Figure 3. A pathway analysis of the top 6,000 differentially expressed genes (DEGs) between SZhigh and SZlow groups. A bar plot shows the top 30 pathways that the DEGs were enriched in. The upregulated pathways in SZhigh group are shown in red, while the downregulated pathways in SZhigh group are shown in blue. DEGs were significantly enriched in pathway related to multiple sclerosis (highlighted in bold). SZhigh group, schizophrenia with high *AQP4* expression; SZlow, schizophrenia with low *AQP4* expression.

Supplementary Data

Anonymized processed source data underlying Supplementary Figures 1–3 are provided in the accompanying Excel file.

Supplementary Table 1. Source data underlying Supplementary Figures 1 and 2.

This table provides anonymized AQP4 mRNA values, GFAP protein values in the prefrontal cortex, and SZhigh/SZlow group labels used for Supplementary Figures 1 and 2. “NA” indicates that the GFAP protein value was not available.

Supplementary Table 2. Processed DEG list underlying Supplementary Figure 3.

This table provides the processed DEG list used as input for Ingenuity Pathway Analysis (IPA). The column “logFC_SZhigh_vs_SZlow” corresponds to the original logFC value from the edgeR output; positive values indicate higher expression in the SZhigh group relative to the SZlow group.

Supplementary Table 3. IPA canonical pathway output underlying Supplementary Figure 3.

This table provides the canonical pathway output from IPA, including pathway-level statistics and associated molecules, where available.

**References:**

1.	Shimamoto-Mitsuyama C, Nakaya A, Esaki K, Balan S, Iwayama Y, Ohnishi T, et al. Lipid pathology of the corpus callosum in schizophrenia and the potential role of abnormal gene regulatory networks with reduced microglial marker expression. Cereb Cortex N Y N 1991. 2021;31:448–62.

2.	Kunii Y, Matsumoto J, Izumi R, et al. Evidence of altered phosphoinositide signaling-associated molecules in the postmortem prefrontal cortex of patients with schizophrenia. Int J Mol Sci. 2021;22:8280.

3.	Miyahara K, Hino M, Shishido R, et al. Identification of schizophrenia symptom-related gene modules using post-mortem brain transcriptome analysis. Transl Psychiatry. 2023;13:144.

4.	Robinson MD, McCarthy DJ, Smyth GK. edgeR: Bioconductor package for differential expression analysis of digital gene expression data. Bioinformatics. 2010;26:139-40.

5.	Krämer A, Green J, Pollard J Jr, Tugendreich S. Causal analysis approaches in Ingenuity Pathway Analysis. Bioinformatics. 2014;30:523-30.

6.	Nagaoka A, Hino M, Izumi R, Shishido R, Ishibashi M, Hatano M, et al. Availability of individual proteins for quantitative analysis in postmortem brains preserved in two different brain banks. Neuropsychopharmacol Rep. 2024;44:399-409.
